# How Echocardiographic Deformation Indices Can Distinguish Different Types of Left Ventricular Hypertrophy

**DOI:** 10.5935/abc.20180223

**Published:** 2018-11

**Authors:** José Luiz Barros Pena, Wander Costa Santos, Stanley de Almeida Araújo, Glauber Monteiro Dias, Eduardo Back Sternick

**Affiliations:** 1 Pós-Graduação Ciências Médicas - Faculdade de Ciências Médicas de Minas Gerais, Belo Horizonte, MG - Brazil; 2 Hospital Felício Rocho, Belo Horizonte, MG - Brazil; 3 Universidade Federal de Ouro Preto (UFOP), Belo Horizonte, MG - Brazil; 4 Instituto Nacional de Cardiologia (INC), Rio de Janeiro, RJ - Brazil

**Keywords:** Speckle Tracking, PRKAG2 Mutation, Deformation Indices, Left Ventricular Hypertrophy, Cardiomegaly, Echocardiography/diagnosis

We present cases of athlete’s heart, idiopathic HCM, and glycogen storage cardiomyopathy (PRKAG2).

The two non-athlete patients (Pts) underwent genetic studies and myocardial biopsies.

Echocardiography showed moderate to severe LVH in all cases.

**Figure 1:**
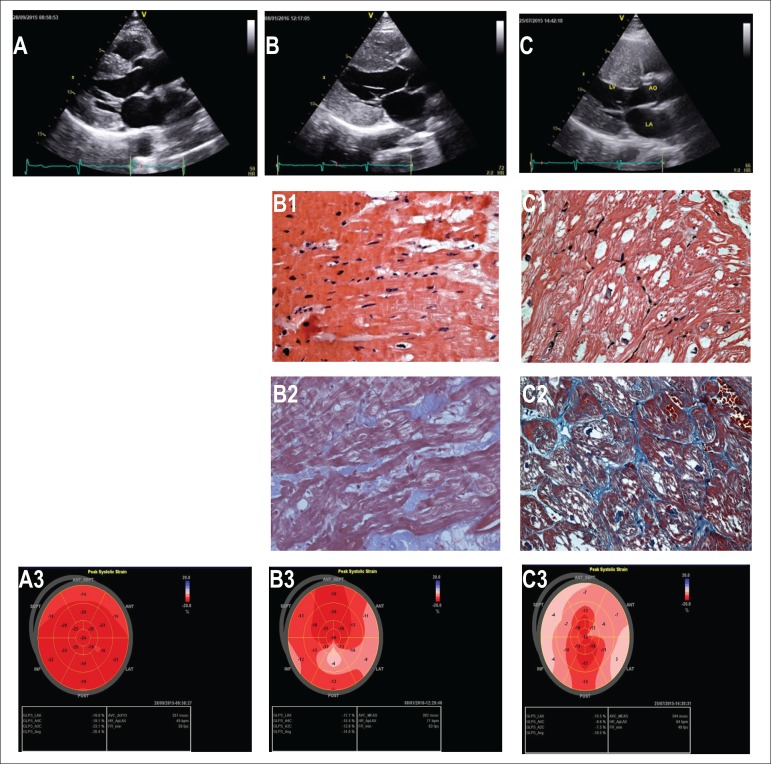
Two-dimensional echocardiography (A, B, C), endomyocardial biopsies (B1, B2, C1, C2) and bullseye maps (A3, B3, C3).

**Case 1,** Figure 1-A**,** A-3 - Athlete, male, 26, intense exercise practice. Automated function imaging: 2D LV strain bullseye map showing normal Longitudinal Regional Myocardial Deformation (LRMD), despite LVH. GLS (global longitudinal strain) -20.4%.

**Case 2,** Figure 1-B**,** B-1**,** B-2**,** B-3 - Male, 26, tachycardia and palpitations with myosin essential light chain 3 mutation. LRMD is typically reduced where hypertrophy is more accentuated. GLS -14.0%.

Figure B-1 - Section of RV ventricular myocardium in HCM, demonstrating marked myocyte hypertrophy and disorganization (HE-stained).

Figure B-2 - Gomoritrichrome stain (GS) showing intense fibrosis in extracellular matrix (blue) and cardiomyocyte architecture disarray.

**Case 3,** Figure 1-C**,** C-1**,** C-2**,** C-3 - Male, 22, palpitations and tachycardia. Genetic analysis found missense mutation, a heterozygous pathogenic variant for PRKAG2 c.905g > A p.(Arg302Gln). LRMD shows deformation levels in a striped pattern. GLS -10.5%.

Figure C1 - HE-stained RV showing myofiber vacuolization with gross granular glycogen inclusions within vacuoles, without cardiomyocyte architecture disarray.

Figure C2-GS showing intense myofiber vacuolization (white) and extracellular matrix collagen fibers without fibrosis (blue).

STE (speckle tracking echocardiography) differentiates LVH and infiltrative disorders. We tried to instantaneously identify disease-related patterns.

To our knowledge, we present the first pattern in a PRKAG2 mutation Pt bullseye map, differentiated from other causes of LVH.^1^ We recommend GLS polar map analysis to improve accuracy in echocardiographic examinations involving moderate LVH. STE can suggest the etiology, critically important to improve therapeutic strategies.
